# The Accuracy of ChatGPT-4o in Interpreting Chest and Abdominal X-Ray Images

**DOI:** 10.3390/jpm15050194

**Published:** 2025-05-10

**Authors:** Pietro G. Lacaita, Malik Galijasevic, Michael Swoboda, Leonhard Gruber, Yannick Scharll, Fabian Barbieri, Gerlig Widmann, Gudrun M. Feuchtner

**Affiliations:** 1 Department Radiology, Innsbruck Medical University, 6020 Innsbruck, Austriamalik.galijasevic@i-med.ac.at (M.G.); michael.swoboda@i-med.ac.at (M.S.); yannick.scharll@i-med.ac.at (Y.S.); gerlig.widmann@i-med.ac.at (G.W.); 2 Department of Cardiology, Angiology and Intensive Care Medicine, Deutsches Herzzentrum Charite, 10117 Berlin, Germany

**Keywords:** artificial intelligence (AI), large language model (LLM), chest X-ray (CXR), abdominal X-ray (AXR)

## Abstract

**Background/Objectives:** Large language models (LLMs), such as ChatGPT, have emerged as potential clinical support tools to enhance precision in personalized patient care, but their reliability in radiological image interpretation remains uncertain. The primary aim of our study was to evaluate the diagnostic accuracy of ChatGPT-4o in interpreting chest X-rays (CXRs) and abdominal X-rays (AXRs) by comparing its performance to expert radiology findings, whilst secondary aims were diagnostic confidence and patient safety. **Methods**: A total of 500 X-rays, including 257 CXR (51.4%) and 243 AXR (48.5%), were analyzed. Diagnoses made by ChatGPT-4o were compared to expert interpretations. Confidence scores (1–4) were assigned and responses were evaluated for patient safety. **Results:** ChatGPT-4o correctly identified 345 of 500 (69%) pathologies (95% CI: 64.81–72.9). For AXRs 175 of 243 (72.02%) pathologies were correctly diagnosed (95% CI: 66.06–77.28), while for CXRs 170 of 257 (66.15%) were accurate (95% CI: 60.16–71.66). The highest detection rates among CXRs were observed for pulmonary edema, tumor, pneumonia, pleural effusion, cardiomegaly, and emphysema, and lower rates were observed for pneumothorax, rib fractures, and enlarged mediastinum. AXR performance was highest for intestinal obstruction and foreign bodies, and weaker for pneumoperitoneum, renal calculi, and diverticulitis. Confidence scores were higher for AXRs (mean 3.45 ± 1.1) than CXRs (mean 2.48 ± 1.45). All responses (100%) were considered to be safe for the patient. Interobserver agreement was high (kappa = 0.920), and reliability (second prompt) was moderate (kappa = 0.750). **Conclusions:** ChatGPT-4o demonstrated moderate accuracy for the interpretation of X-rays, being higher for AXRs compared to CXRs. Improvements are required for its use as efficient clinical support tool.

## 1. Introduction

Personalized treatment of diseases plays a pivotal role in enhancing the effectiveness and precision of therapeutic interventions in modern clinical practice. By tailoring medical decisions, practices, and interventions to individual patients based on their unique clinical characteristics, personalized medicine seeks to move beyond the traditional “one-size-fits-all” approach. This approach depends heavily on an accurate diagnosis of the underlying pathology driving disease processes, which is essential for selecting the most appropriate and effective therapies for each patient. Radiographic imaging, particularly chest X-rays (CXRs) and abdominal X-rays (AXRs), is a cornerstone of medical diagnostics. In the context of personalized medicine, these imaging techniques serve as vital tools for early and accurate disease diagnosis and treatment monitoring, enabling clinicians to customize interventions based on the patient’s specific radiographic presentation and clinical trajectory. These imaging techniques are effective in detecting specific pathological changes while offering the advantages of being noninvasive and economically viable [1]. General practitioners or physicians who do not specialize in radiology often seek prompt diagnostic insights without waiting for formal radiology reports, and AI tools offer the unique opportunity to assist the preliminary interpretation of radiological images, particularly in resource-limited or fast-paced settings, to accelerate the delivery of personalized care through faster triage and more timely clinical decisions. X-rays are among the most frequently used imaging modalities, especially in general practice and intensive care units [2], for diagnosing common thoracic and abdominal pathologies, including pneumonia, pneumothorax, and bowel obstruction.

Additionally, an increasing number of patients nowadays have direct access to their imaging studies through cloud-based online repositories provided by healthcare institutions. Their curiosity about understanding these images should not be underestimated, as many actively seek more information about their diagnoses. However, the extent to which the information available to patients is reasonable and safe has not been thoroughly evaluated.

The growing accessibility of multimodal large language models (LLMs), such as ChatGPT-4 Vision (GPT-4V), highlights their potential utility in direct image interpretation—particularly for non-radiologists or in settings where immediate radiological expertise is unavailable. This approach could be especially beneficial for other clinicians as radiological examinations are sometimes requested with incomplete or inadequate patient history and clinical indications [3,4]. This leads to an uncertain preliminary report that requires further discussion, resulting in a subsequent delay in finalizing the formal report. By accelerating triage processes and contributing to faster clinical decisions, such tools may facilitate more rapid deployment of patient-specific care plans, ultimately improving outcomes through earlier, targeted interventions.

Unlike traditional LLMs optimized solely for text generation, multimodal models like GPT-4V integrate computer vision capabilities, allowing them to analyze images and generate diagnostic hypotheses [5,6]. In the field of radiology, several studies have investigated the potential of these models to improve diagnostic efficiency and accuracy [7,8,9,10,11,12,13,14,15,16].

However, their reliability and clinical applicability must be rigorously evaluated against expert radiologists, as highlighted in prior research [17,18,19].

In theory, such models could assist with initial triage or provide decision support by identifying obvious pathologies or suggesting appropriate follow-up examinations and downstream testing, thereby expanding the potential applications of LLMs in both clinical radiology [20,21] and research [22]. Consequently, ChatGPT may serve as a valuable clinical support tool for non-radiologist physicians—for example, those working in emergency departments, intensive care units, or primary care. Therefore, the primary aim of this study was to assess the diagnostic performance of ChatGPT in detecting common pathologies in basic radiological cases (CXR and AXR images) relevant to the acute care setting, in comparison to diagnosis made by expert radiologists. As a secondary objective, we evaluated ChatGPT’s diagnostic confidence and whether its responses could be considered “safe” for patients—defined as not causing unnecessary concern, fear, or confusion.

This manuscript consists of the following chapters: 2. Material and Methods, 3. Results, 4. Discussion, and 5. Conclusions.

## 2. Materials and Methods

### 2.1. Study Design

This was a retrospective analysis of 500 radiographic cases. The study adheres to the checklist for artificial intelligence in medical imaging [23] and was exempt from institutional review board review due to the use of publicly available data. The dataset consisted of 257 thoracic X-rays (CXRs) and 243 abdominal X-rays (AXRs). Cases were selected to represent a diverse set of common pathologies for each radiological modality, with a focus on acute pathologies which are representative for emergency department (ED) and intensive care (ICU) settings.

### 2.2. Data Collection

The images were sourced from various clinical cases available on the Radiopaedia.org platform, ensuring a broad range of conditions for comparison. To enhance diagnostic clarity, images with clear radiological features were prioritized. For instance, lung tumors larger than 3 cm were selected as these are more easily identifiable on X-ray images. This approach aimed to ensure the inclusion of pathologies with prominent, visible features, thereby facilitating a more accurate assessment of ChatGPT’s diagnostic performance. The pathologies under review for CXR included pneumonia, lung edema, pleural effusion, lung tumors, emphysema, cardiomegaly, enlarged mediastinum, rib fractures, and pneumothorax. For AXRs, the study focused on detecting bowel obstruction, bowel perforations, and renal calculi.

### 2.3. AI-Based Image Analysis

ChatGPT-4o, a large language model (LLM), was assessed for its capability to interpret radiological findings by analyzing submitted case images. The latest version of ChatGPT-4o ((version 4 omni), OpenAI, San Francisco, CA 94158,USA) was accessed and utilized from 10 September 2024 to 10 December 2024 for the first batch of 400 patients and until 31 January 2025 for the second batch of 100 patients to test whether it could provide useful diagnostic support based on uploaded chest or abdominal X-ray images. After each image dataset of 30 patients, a time delay of a minimum of 3 h was set.

Despite lacking specialized training in radiology, ChatGPT’s performance was assessed on its ability to recognize pathology from a single image without access to the patient’s medical history. The model was presented with the following questions (“prompt” #1), “What is going on?” Then, one experienced radiologist with 5 years of training conducted the analysis and proof-checked the diagnosis of radiopedia.org in the time between downloading from the platform and uploading to ChatGPT. Furthermore, the difficulty of the pathologies was scored by a radiologist with 5 years of training (board-equivalent) at either level 1 = easy or level 2 = difficult. The same rating was repeated by a board-certified radiologist with >10 years of training, and the interobserver rating was calculated with a weighted Cohen’s Kappa.

**#1 Primary analysis:** The results of ChatGPT’s interpretations were compared with the diagnoses from radiopedia database cases. Each diagnosis was rated as either correct (“yes”) or incorrect (“no”) based on the match between ChatGPT’s output and the expert radiologist’s diagnosis. Performance metrics, including sensitivity, specificity, and overall accuracy, were calculated.

Confidence score were graded on a 4-point scale: 4 = high (very confident, this is a pneumothorax), 3 = moderate (this is very like likely, probably), 2 = low (uncertain, vague) but still correct (this could be, maybe is a pneumothorax), 1 = very low (very vague, e.g., this could be, but it could also be something else), and 0 = incorrect diagnosis. The rating was made by the same observer. This analysis was repeated by a second independent observer, also a board-certified radiologist, who was blinded to the results of the 1st observer. A subset of 100 questions was used for the calculation of the interobserver variability.

Reliability (“repeatability”) analysis: The same query was repeated after 3 months, with the same (prompt #1) and the same observer evaluated the correctness of the answers for (“intraobserver variability”).

**#2 Secondary analysis:** ChatGPT was asked the most common patient question (prompt: “What shall I do now”?). The answers of ChatGPT for both question #1 and #2 were evaluated for patient interpretability and their reaction, with 1= safe (reasonable and not concerning) and 0 = concerning. “Concerning” was defined as triggering an unnecessary emotional response such as concern and fear, or through creating confusion (for example, by providing too much threatful though correct data (e.g., the risk of death is very high)) by one observer.

**Figure 1:**
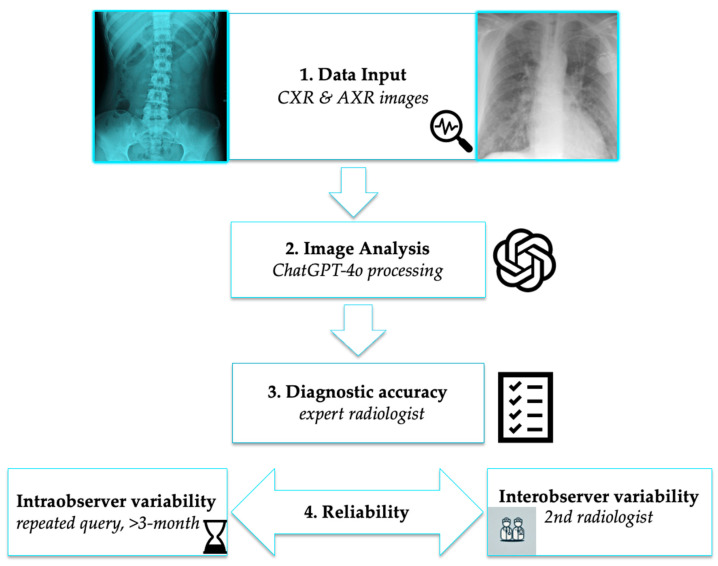
Workflow for evaluating ChatGPT-4o in radiographic interpretation. The process includes data selection, AI-based image analysis, expert validation, and repeatability assessment. Underlined text in the figure represents key decision points.

The entire image evaluation process is illustrated at Figure 1.

#### Statistical Analysis

The diagnostic accuracy was calculated using an open access statistical platform (openepi.com). Descriptive statistics were calculated with SSPS (IBM). Normality of data distribution was tested with a histogram and the Shapiro–Wilk test. Parametric data are shown as mean ± SD and non-parametric data as median (interquartile range, IQR). Categorical data are presented as N (counts) and % (percentage). A Chi-square test was applied to test for differences in groups with categorical data. For reliability analysis, interobserver variability was calculated with Cohen’s weighted Kappa, and the 2nd query after 3 months (“intraobserver variability”) was used.

## 3. Results

### Diagnostic Accuracy

ChatGPT successfully responded to all 500 queries (100%). The dataset included 257 CXRs (51.4%) and 243 AXRs (48.5%). The diagnostic accuracy in interpreting 500 X-rays was 345 out of 500 (69%) pathologies correctly identified (95% CI: 64.81–72.90). The diagnostic accuracy for AXR was 175 out of 243 (72.02%) correctly identified pathologies (95% CI: 66.06–77.28), while for CXR 170 out of 257 (66.15%) pathologies were correctly diagnosed (95% CI: 60.16–71.66). The results are shown in Table 1. Patient age was mean 45.05 +/− 24.7 years (range, 1–92 years) and 35.7% were women. Both pediatric and adult cases were included.

The accuracy for the different CXR and AXR pathologies, their detection rates, and the confidence scores are shown in Table 1. The mean confidence score was 2.95 ± 1.37 (median 4 (IQR 2). For CXR, the mean confidence score was 2.48 ± 1.45 and median was 4 (IQR 3), and for AXR, the mean was 3.45 ± 1.1 SD and the median was 4 (IQR 1).

**Figure 2:**
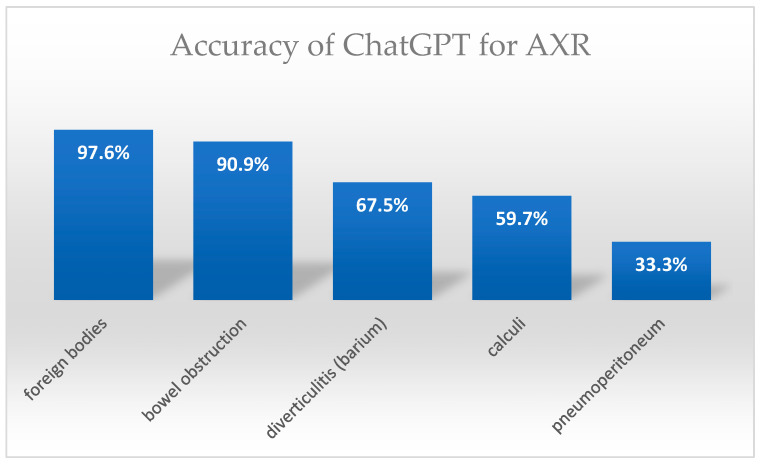
Accuracy of ChatGPT in diagnosing different abdominal X-ray (AXR) pathologies. AXR performance was highest for intestinal obstruction and foreign bodies but weaker for pneumoperitoneum, renal calculi, and diverticulitis.

For CXRs the detection rate of pneumonia was 74%, lung edema was 100%, pleural effusion was 70%, lung tumors was 90%, emphysema was 81.8%, cardiomegaly was 72.7%, and enlarged mediastinum was 54.5%. None of the rib fractures (0%) were detected. (Figure 2). For pneumothorax, the detection rate was 21 of 51 (41.2%).

**Figure 3:**
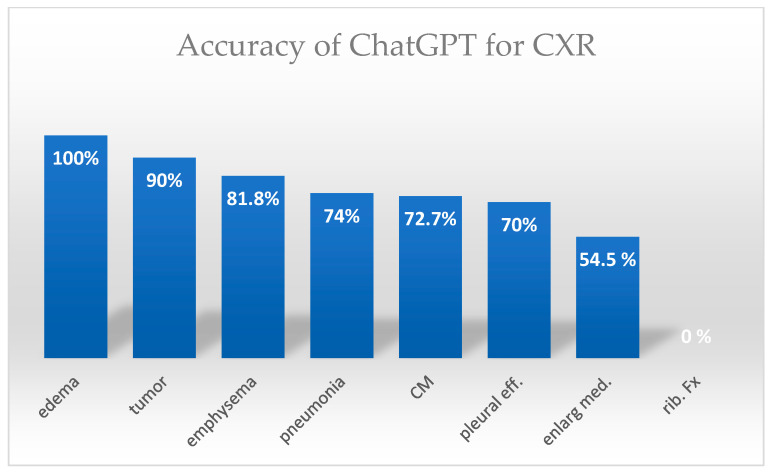
Accuracy of ChatGPT in diagnosing different chest X-ray (CXR) pathologies. CXR diagnoses showed high detection rates for pulmonary edema, tumor, emphysema, pneumonia, cardiomegaly, and pleural effusion, but lower accuracy for pneumothorax, enlarged mediastinum, and rib fractures. Abbreviations: CM = cardiomegaly. Med. = mediastinum. Fx = fracture. Eff = effusion.

The accuracy of AXR was 90.9% for bowel obstruction (both small and large bowel), 33.3% for pneumoperitoneum, 59.7% for calculi (renal, ureteral, bladder, and gallbladder), 67.5% for diverticulitis (barium study), and 97.6% for foreign bodies (Figure 3).

The levels of difficulty for the X-rays were as follows: 107 (21.1%) difficult and 394 (78.8%) easy. The accuracy of ChatGPT in difficult cases was significantly lower (50/106; 47.1% correct diagnosis rate) than for easy cases (295/394, 74.87%) (Chi-square, *p* < 0.001). Interobserver agreement between a radiologist with 5 years of training (board equivalent) and a board-certified radiologist with 10 years of training was very high with a weighted Cohen Kappa = 0.938 (95% CI 0.900–0.976; *p* < 0.001)

**Figure 4:**
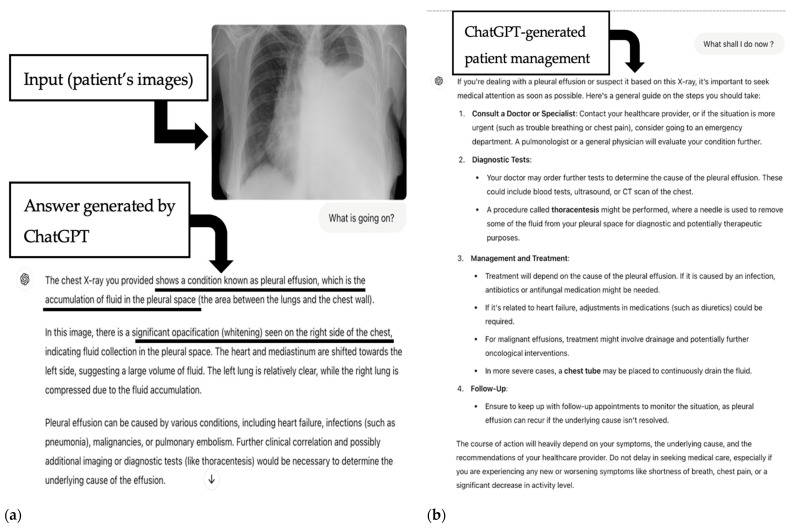
Example of a patient chest X-ray analyzed by ChatGPT-4o. (**a**) Input (patient’s images) to ChatGPT, with text generated by ChatGPT and the correct diagnosis (pleural effusion, underlined in black). (**b**) Suggested next steps in patient management as generated by ChatGPT. Upper right image (a) (case courtesy of Dr Ian Bickle, Radiopaedia.org rID 26730).

**Figure 5:**
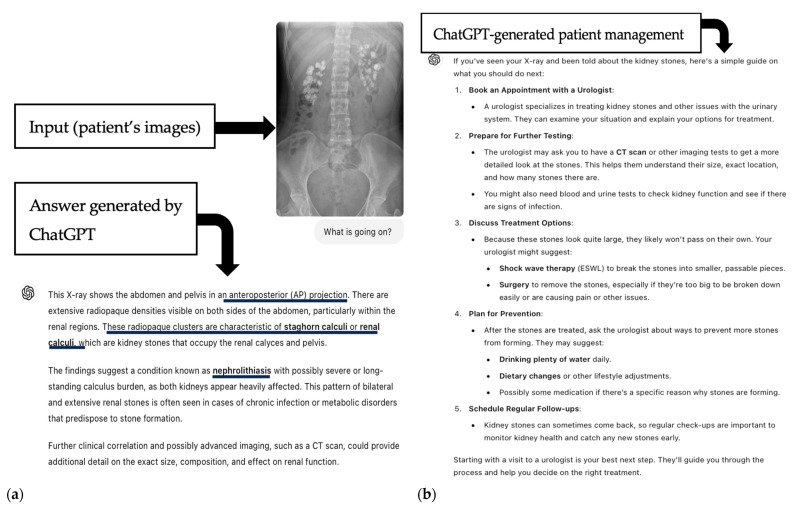
Example of an abdominal X-ray analyzed by ChatGPT-4o. (**a**) Input to ChatGPT, with text generated by ChatGPT and the correct diagnosis (renal calculi, underlined in black). (**b**) Next steps in patient management as provided by ChatGPT. Upper right image (**a**) (case courtesy of Dr Vikas Shah, Radiopaedia.org rID 164910).

Safety: The answers provided were 100% safe (500/500). None of the questions were subjectively rated as triggering unnecessary fear, concerns, or confusion. Examples of ChatGPT’s responses are presented in Figure 4 and Figure 5.

Reliability: The interobserver variability was weighted kappa = 0.920 +/− 0.039 SD (95% CI: 0.844–0.997, *p* < 0.001). Intraobserver variability (second query after 3 months) was conducted in a cohort of 100 patients. The repeatability (“intraobserver variability”) was moderate with kappa = 0.750 +/− 0.119 SD (95% CI 0.517–0.983, *p* < 0.001).

## 4. Discussion

The results of this study provide valuable insights into the potential and limitations of large language models (LLMs) like ChatGPT and their respective visual modules for image analysis in the field of radiology. Overall, ChatGPT-4o demonstrated a moderate diagnostic accuracy when interpreting conventional abdominal and thoracic images, achieving a 69% success rate across 500 cases. Its performance varied across different radiological modalities, with a higher accuracy for abdominal X-rays (AXRs) (72.02%) compared to chest X-rays (CXRs) (66.15%).

Large language models (LLMs) are gaining increasing popularity in the medical domain due to their capacity to serve as clinical support tools for various tasks, such as answering specific medical queries, suggesting therapeutic options, and assisting in the selection of imaging protocols [24]. Furthermore, LLMs have been evaluated for their potential in interpreting medical imaging, such as computed tomography angiograms (CTAs) [25], and in proposing differential or final diagnoses based on radiological reports [26]. This interest has grown, especially after the release of ChatGPT-4Vision in October 2023 which introduced the possibility to generate a description of image content without introducing additional information [27]. This novel algorithm is helpful for translating complex medical reports into clearer, more accessible language, thereby enhancing patient understanding [13,28]. Moreover, it shows promising potential in supporting radiological decision-making [29,30].

In this context, ChatGPT may also serve as an enabler of personalized medicine. Its ability to rapidly identify common and urgent pathologies can facilitate early patient stratification and more targeted follow-up. Providing timely diagnostic insights to physicians across various clinical settings—even before a formal radiology report is available—has the potential to significantly enhance both personalized and precision medicine. By enabling earlier recognition of the underlying pathology, AI tools like ChatGPT-4o may support the prompt initiation or adjustment of therapeutic interventions tailored to the specific clinical condition of each patient. For instance, in cases of acute dyspnea—where potential causes include pneumothorax, pneumonia, or pleural effusion—a rapid and accurate preliminary diagnosis based on X-ray findings may enable more immediate and individualized treatment decisions. The delays inherent in awaiting formal radiological assessments can hinder timely care, particularly in emergency and acute care settings, and AI-assisted image interpretation may help bridge this gap.

Artificial intelligence has seen significant adoption in radiology over the past year, driven by its numerous advantages and its transformative potential to support personalized medicine [31]. Key machine learning techniques include convolutional neural networks (CNNs) and algorithms such as gradient boosting and support vector machines (SVMs), which are particularly suited for image analysis and pattern recognition and are therefore more commonly applied in radiology.

A landmark contribution to the field is the multicenter study by Sim et al. [32], published in 2019, which demonstrated that deep learning-assisted radiologists achieved greater sensitivity in detecting malignant lung nodules on chest X-rays with greater sensitivity and a reduced false positive rate, regardless of reader experience or imaging systems. This ability to augment diagnostic performance through AI represents a crucial step toward more personalized diagnostics, ensuring that subtle differences in disease features—potentially overlooked by human readers—are identified earlier and more consistently.

Several subsequent studies have explored the application of deep CNN-based systems for the detection of pathologies on chest X-rays [32,33,34,35,36,37,38]. For example, Castiglioni et al. [32] demonstrated that machine learning applied to CXRs significantly enhances diagnostic support for the diagnosis of COVID-19 by improving the diagnostic accuracy for COVID-19 compared to traditional visual read-outs by radiologists. This capability to implement learned patterns across large, heterogeneous patient datasets supports more precise, patient-specific diagnosis potentially affecting treatment decisions, aligning AI applications in radiology with the core objectives of personalized medicine.

More recently, Weiss et al. [39] reported the prediction of the 10-year cardiovascular risk for major adverse cardiovascular events (MACEs) directly from CXRs using an advanced deep learning model, comparing its performance to the traditional ASCVD (atherosclerotic cardiovascular disease) risk score. The model was validated on over 11,000 patients, highlighting its potential as a novel tool for cardiovascular risk assessment [39], offering a novel approach for individualized risk stratification based on routinely available imaging, with the potential to enhance the precision of personalized therapies for coronary artery disease prevention (for example lipid-lowering medication such as statins).

However, ChatGPT has not been validated extensively regarding its ability to interpret radiological images. This limitation arises because ChatGPT is optimized for text-based applications, and other models specifically designed for image analysis—such as CNNs, support vector machines, or gradient boost algorithms—are more commonly employed in radiological machine learning tasks.

In our study, for CXRs, ChatGPT was particularly effective in identifying more straightforward pathologies, such as pneumonia and cardiomegaly. However, its performance declined notably in cases involving conditions like pleural effusion, enlarged mediastinum, and pneumothorax, which often present with more subtle or overlapping radiographic features. Furthermore, the detection of rib fractures was very poor with none (0%) identified correctly. This reduction in diagnostic performance may be attributed to the inherent complexity of thoracic imaging, where pathological findings can vary considerably depending on patient-specific factors such as positioning, co-existing conditions, and variations in radiographic technique. Additionally, certain pathologies are difficult to visualize due to their small lesion size or minimal contrast differences relative to surrounding tissues. Even experienced radiologists may encounter diagnostic challenges in these scenarios, which could partly explain the limitations observed in ChatGPT’s performance.

In the evaluation of AXRs, ChatGPT exhibited higher accuracy compared to CXRs and a moderate accuracy in detecting bowel obstructions and foreign bodies—pathologies characterized by distinct and well-defined radiographic features and greater contrast attenuation relative to adjacent tissue. The accuracy for calculi (renal, ureteral, and gallbladder) was rather poor, with approximately only half of such cases correctly identified, again underscoring the challenge AI models face in detecting more nuanced subtle imaging findings which are required for optimized individualized patient care.

ChatGPT also struggled with more complex cases, such as bowel perforations, where the presence of free air under the diaphragm can be subtle, easily confused with other signs, or obscured by superimposed structures and lines. The diagnostic performance for pneumoperitoneum was poor, with only 33.3% of cases correctly detected. This highlights an ongoing challenge for AI models—while they perform relatively well in identifying well-defined patterns their reliability diminishes when confronted with less distinct, multifactorial presentations, small lesions, and overlapping anatomical structures

The higher accuracy observed in AXR cases, relative to CXR cases, may be attributed to the more specific radiographic features of certain abdominal pathologies and ChatGPT’s predisposition to recognize prominent, clear-cut findings.

The overall accuracy of ChatGPT (69%) indicates that it is not yet capable of consistently matching the nuanced decision-making skills of human radiologists, particularly in more complex cases. Its failure rate is significant enough to warrant caution against deploying such models without human oversight. However, ChatGPT correctly diagnosed approximately about two thirds of cases which highlights its promising potential, particularly for routine cases and those involving clear, easily identifiable radiological findings.

The integration of AI into radiology has been proposed as a tool to enhance diagnostic workflows, increase efficiency, and potentially reduce errors, particularly in high-volume clinical settings. ChatGPT’s relatively high accuracy in simpler cases suggests that it could serve as valuable clinical support tool for non-radiologists working in emergency departments or intensive care units. It could function as a triage tool, flagging obvious pathologies for expedited radiological review while filtering out normal cases. This approach could help alleviate the workload of radiologists, allowing them to focus on more complex cases that require nuanced interpretation.

Nonetheless, the potential of AI to support personalized care pathways remains compelling. ChatGPT, even with its limitations, could serve as a frontline triage tool in settings with limited radiologist availability, such as rural or underserved regions. Also, ChatGPT could assist patients by explaining their condition in a clear and reasonable manner, potentially reducing the need for additional consultations or second opinions.

Despite these potential benefits, the discrepancies in performance between ChatGPT and professional radiologists underscore the need for human expertise in the diagnostic decision-making process. Radiologists are trained to consider the clinical context of each case, the quality of the image, artifacts, and subtle variations in imaging findings that may not be easily recognized by LLMs. In many cases where ChatGPT failed to provide an accurate diagnosis, subtle findings with lower contrast attenuation differences, small-sized lesions, or complex anatomical factors such as overlapping organs likely contributed to errors. Additionally, radiologists employ critical thinking and a degree of skepticism when interpreting ambiguous or unclear findings and are better trained to distinguish image artifacts from true pathologies. The latter task remains one of the greatest challenges for AI-based tools as artifacts can vary significantly in their appearance among different patients and can frequently mimic or even obscure true pathological findings. Furthermore, humans can adjust their interpretation based on the patient’s history, clinical presentation, and additional imaging or laboratory results, providing significant advantage over AI models that rely solely on pattern recognition. While ChatGPT demonstrates impressive capabilities in recognizing common radiological patterns, it lacks the ability to integrate broader clinical insights, making it more prone to errors in complex cases where a comprehensive understanding of the patient’s condition is essential.

However, the use of ChatGPT as standalone diagnostic decision-making tool is currently not permitted. It must be emphasized that Chat GPT is not a certified medical product according to international regulations, with no Federal Drug Agency (FDA) approval in the US, and no European Medical Agency (EMA) (“CE-mark”) approval, hence its use for decision-making is not allowed. However, ChatGPT may act as a useful “clinical support” tool. It should be employed as a complementary tool that assists radiologists by providing a second opinion or by highlighting areas of concern that may warrant further review.

Regulatory and ethical considerations play a fundamental role in the future of AI in radiology. Compliance with data protection regulations, transparent validation protocols, and ongoing model monitoring are essential to ensure patient safety and maintain trust [40]. Ethical concerns, such as avoiding automation bias and maintaining clinicians’ role as the primary diagnosticians, will also shape how these tools are implemented and accepted in clinical environments.

ChatGPT has been recently investigated in various fields of medicine, including clinical report interpretation [28], oncology [29], musculoskeletal radiology [19], or in passing medical exams such as the USMLE [41]. In determining the correct diagnosis based on a patient’s clinical history, ChatGPT has recently shown a promising potential and added value to physician-made diagnosis in a single-blinded trial involving a small series (n = 50) of patients [42], although the availability of an LLM to physicians as a diagnostic aid did not significantly improve clinical reasoning compared with conventional resources. The LLM alone demonstrated higher performance than both physician groups, indicating the need for technology and workforce development to realize the potential of physician–artificial intelligence collaboration in clinical practice. Prior studies highlight the superior capability of LLM in text-based language processing compared to image texture analysis.

This study has several limitations. ChatGPT is fundamentally a language model designed primarily for text-based tasks rather than for image analysis. Without specific training in medical imaging, its diagnostic accuracy is likely limited compared to models developed explicitly for radiology, such as convolutional neural networks (CNNs) or other image-specific machine learning systems. An additional limitation is the variability in responses generated by large language models (LLMs), which has been reported in previous studies. The tendency of ChatGPT to produce different answers to the same query at different times highlights the need for structured prompt engineering to minimize inconsistencies and ensure reproducibility of results [43]. To avoid a deterioration of responses, we have stretched the entire queries over an extended period of 4 months, and a time delay of a minimum of 3 h was applied between batches of n = 30 images.

Furthermore, the model was evaluated solely on X-ray images, without access to the clinical context, such as patient history or presenting symptoms, which often provides incremental value to image interpretation. The absence of this contextual information may have limited the model’s ability to generate more accurate diagnoses.

The study also focused on common thoracic and abdominal pathologies, which may not fully reflect the complexity and variability encountered in actual clinical cases. Finally, we tested only a single LLM (Chat GPT-4o) without comparison to other versions or models such as Llama or Gemini. The performance of different models is variable [44,45]. Gunes et al. [44] found a high variability among the 124 chest cases with the highest accuracy for Claude 3 Opus (70.29%), followed by ChatGPT 4/Google Gemini 1.5 Pro (59.75%), and Meta Llama 3 70b (57.3%), whilst the accuracy for an older version, ChatGPT 3.5 (53.2%), was lower. Another study by Arnold et al. (4%) investigated different LLMs for their performance of accurately assigning the standardized clinical reporting system (CADRADS 2.0) from non-uniform various clinical CT reports and found major differences between the LLMs. While GPT-4o and Llama3 70b achieved the highest accuracy with a performance rate of 93% and 92.5%, older LLMs (Mixtral 7b and GPT-3.5) performed poorly with an accuracy of 16% [45].

The concept of “safety” for the patient in this study was defined as the absence of inducing unnecessary concerns, fear, or confusion for patients. However, this subjective approach may not fully capture the broader range of potentially misleading or confusing information that AI responses could generate. Moreover, the inherent variability in subjective evaluations represents another limitation of the study. Furthermore, as this study assessed the capabilities of a single model iteration, ChatGPT-4o, these findings may not generalize to future versions or other multimodal LLMs as these may have enhanced image interpretation capabilities. Future studies should compare other LLM types (e.g., Llama, Mixtral, and others) with the one used in our study.

Future Directions: Looking ahead, several key areas offer opportunities in AI models such as ChatGPT. One of the most significant challenges in radiology is the accurate interpretation of complex cases where multiple pathologies or overlapping features may be present. Future iterations of AI models will need to incorporate a more nuanced understanding of radiographic findings and the ability to integrate clinical context to improve diagnostic accuracy.

Additionally, future research should focus on training AI models with larger and more diverse datasets with a broader range of pathologies from clinical routine, including rare and complex cases. Since the performance of AI models is heavily dependent on training data, such diversity will be essential for improving diagnostic capabilities. Ongoing refinement, along with continuous validation through external studies in varied clinical settings, will be critical to building trust in AI-based diagnostics. Furthermore, the applicability of AI models to other imaging modalities, such as computed tomography (CT) and magnetic resonance imaging (MRI), should also be explored. Beyond diagnostics, AI holds significant potential in educational settings by serving as a learning tool for radiology trainees, providing instant feedback and exposing them to a wide variety of cases within a short timeframe.

In addition, the role of AI in answering patients’ questions regarding their medical conditions warrants investigation to identify potential sources of misinformation and mitigate risks. ChatGPT could play a valuable role in patient-centered communication, explaining findings in accessible language that may empower patients to better understand their own health, thereby supporting shared decision-making and individualized health education. This function aligns with the goals of personalized medicine as the patient is seen not just as a case but as a partner in the care process. While ChatGPT’s current diagnostic capabilities fall short of fully replacing expert radiologists, its potential to contribute to augmentative, personalized decision-making frameworks is significant—particularly as models evolve to better incorporate multimodal inputs and contextual data. As these systems mature they may help to reduce diagnostic delays, tailor interventions more precisely, and ultimately contribute to more individualized care plans that reflect each patient’s unique presentation.

Finally, quality assurance monitoring of AI models remains crucial to assess and maintain their accuracy and performance and to identify domains with higher error rates.

Therefore, we conducted a reliability analysis assessing both interobserver variability and repeatability through a second set of identical prompts issued three months later. While interobserver variability was very high, repeatability testing revealed more significant deviations, with a moderate agreement of kappa = 0.750. The deterioration of LLMs over time is a known limitation [46], with models tending to deteriorate after reaching peak performance. For instance, Kouchanek et al. [47] reported an accuracy of only 48–49% for ChatGPT-3.0, with a modest improvement to 65–69% for ChatGPT-4. Finally, continuous quality assurance of LLMs is essential and is, for example, performed by benchmark studies [48]. To further enhance the evaluation of AI-based diagnostic tools, standardized frameworks such as QUADAS-AI and the checklist for artificial intelligence in medical imaging (CLAIM) have been developed [49,50]. These tools aim to ensure methodological rigor, improve reproducibility, and facilitate comparability across studies evaluating AI performance in radiology. Future studies assessing LLMs’ diagnostic capabilities should adopt these frameworks to strengthen the validity of their findings.

## 5. Conclusions

The accuracy of ChatGPT-4o is moderate and not yet sufficient to ensure reliable clinical diagnoses. ChatGPT-4o demonstrated a diagnostic accuracy of 69%, with a higher performance for abdominal X-rays (72.02%) compared to chest X-rays (66.15%). The model exhibited higher accuracy for straightforward and well-defined pathologies, such as pulmonary edema and foreign bodies, but struggled with more complex conditions, such as rib fractures and pneumoperitoneum. While ChatGPT shows potential as a clinical support tool for non-radiologists, especially in settings where immediate radiology expertise is lacking, its current accuracy necessitates cautious use—only under professional supervision, but not as primary diagnostic decision-making tool—which is not permitted in accordance with international medical product regulations. Future developments should prioritize training LLMs with larger, diverse datasets and the integration of patient-specific and multimodal information, paving the way for their incorporation into personalized diagnostic workflows. Additional training has the potential to enhance their overall diagnostic accuracy and the consecutive precision of further tailored personalized therapeutic intervention, and downstream testing. Moreover, ensuring that AI-generated responses are safe, appropriate, and accurate with their information provided to patients is also of great importance for each individual patient.

## Figures and Tables

**Table 1:** Detection rates and confidence scores for different chest X-ray (CXR) and abdominal X-ray (AXR) pathologies as interpreted by ChatGPT-4o. Abbreviations: AXR = abdominal X-ray. CXR = chest X-ray. N = count. IQR = interquartile range. BO= bowel obstruction. R = renal. U = ureter. B = bladder.

CXR Pathology	N	Detection Rate N/N (%)	Confidence Score Median (IQR)	AXR Pathology	N	Detection Rate N/N (%)	Confidence Score Median (IQR)
Pneumonia	50	37/50 (74%)	3 (IQR 2)	Small/Large BO	60	54/60 (90.9%)	3.5 (IQR 2)
Pulmonary edema	30	30/30 (100%)	3 (IQR 0.5)	Pneumoperitoneum	30	10/30 (33.3%)	4 (IQR 0)
Pleural effusion	30	21/30 (70%)	2.5 (IQR 1)	R/U/B calculi or gallstones	73	43/73 (59.7%)	4 (IQR 1)
Lung tumors	10	9/10 (90%)	3.5 (IQR 0.75)	Diverticulitis (Barium)	40	27/40 (67.5%)	4 (IQR 1)
Emphysma	11	9/11 (81.8%)	4 (IQR 1)	Foreign bodies	41	40/41 (97.6%)	4 (IQR 0)
Cardiomegaly	44	32/44 (72.7%)	3 (IQR 2)	**Total**	**243**	**175/243 (72.02%)** 95% CI: 66.06–77.28	**Median 4** **(IQR 1)** **Mean** **3.45 ± 1.1**
Enlarged mediastinum	11	6/11 (54.5%)	3 (IQR 1.25)				
Rib fracture	20	0/20 (0%)	3.5 (IQR 2)				
Pneumothorax	51	21/51 (41.2%)	3 (IQR 1)				
**Total**	**257**	**170/257 (66.15%)** 95%CI:60.16–71.66	**Median 4** **(IQR 3)** **Mean** **2.48 ± 1.45**				

## Data Availability

We do not wish to share our data.

## Abbreviations

The following abbreviations are used in this manuscript:

AI	Artificial Intelligence
LLM	Large Language Model
CXR	Chest X-ray
AXR	Abdominal X-ray
ED	Emergency Department
ICU	Intensive Care Unit
GPT-4V	Generative Pretrained Transformer 4 Vision
GPT-4o	Generative Pretrained Transformer 4 omni
CNN	Convolutional Neural Network
SVM	Support Vector Machine
CTA	Computed Tomography Angiography
BO	Bowel Obstruction
R/U/B	Renal/Ureter/Bladder
CM	Cardiomegaly
Med.	Mediastinum
Fx	Fracture
Eff	Effusion
IQR	Interquartile Range
N	Number (of cases)
SD	Standard Deviation
MACEs	Major Adverse Cardiovascular Events
ASCVD	Atherosclerotic Cardiovascular Disease
USMLE	United States Medical Licensing Examination
